# Apocrine adenocarcinoma of the nipple: a case report

**DOI:** 10.1186/1757-1626-1-88

**Published:** 2008-08-12

**Authors:** George Alex

**Affiliations:** 1 Department of Breast Surgery, Gloucestershire Royal Hospital, Great Western Road, Gloucester, GL1 3NN, UK; 2 Hull Breast Unit, Castle Hill Hospital, Castle Road, Cottingham, East Yorkshire, HU16 5JQ, UK

## Abstract

Apocrine adenocarcinomas are rare malignant skin adnexal tumours. Apocrine carcinoma of the nipple is extremely rare and this case to the author's knowledge is only the third reported case worldwide and the first with associated ductal carcinoma in situ elsewhere in the breast. A seventy one year old caucasian female presented to the breast clinic with a growth on her nipple which proved on histopathological analysis to be an apocrine carcinoma. Recommended treatment for apocrine carcinoma includes surgery in the form of wide local excision.

## Introduction

Cutaneous ductal apocrine adenocarcinomas are extremely rare malignant adnexal tumours [1,2]. Most of these neoplasms involve the axilla. They have been described in other areas as well. The progression of these tumours is quite varied. The standard of care for these lesions consists of wide local excision, with or without lymph node dissection.

## Case presentation

A seventy one year old Caucasian female presented to the breast clinic with a painless growth on her right nipple of six weeks duration. She gave no history of itchiness, discharge or bleeding from the growth. She was otherwise asymptomatic regarding her breasts.

She had previously had no breast problems and had no family history of breast or ovarian carcinoma. She was nulliparous and a teetotaller.

The only medical history of note was severe osteoarthritis of her joints including her shoulder joints.

Clinical findings were threefold.

**Figure 1:**
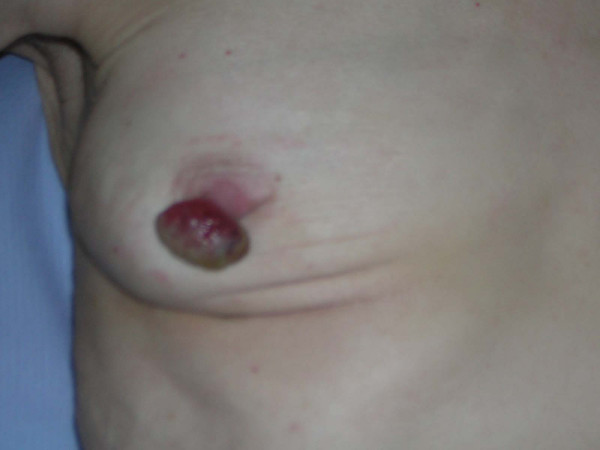
**Apocrine carcinoma of the nipple Image 1**.

**Figure 2:**
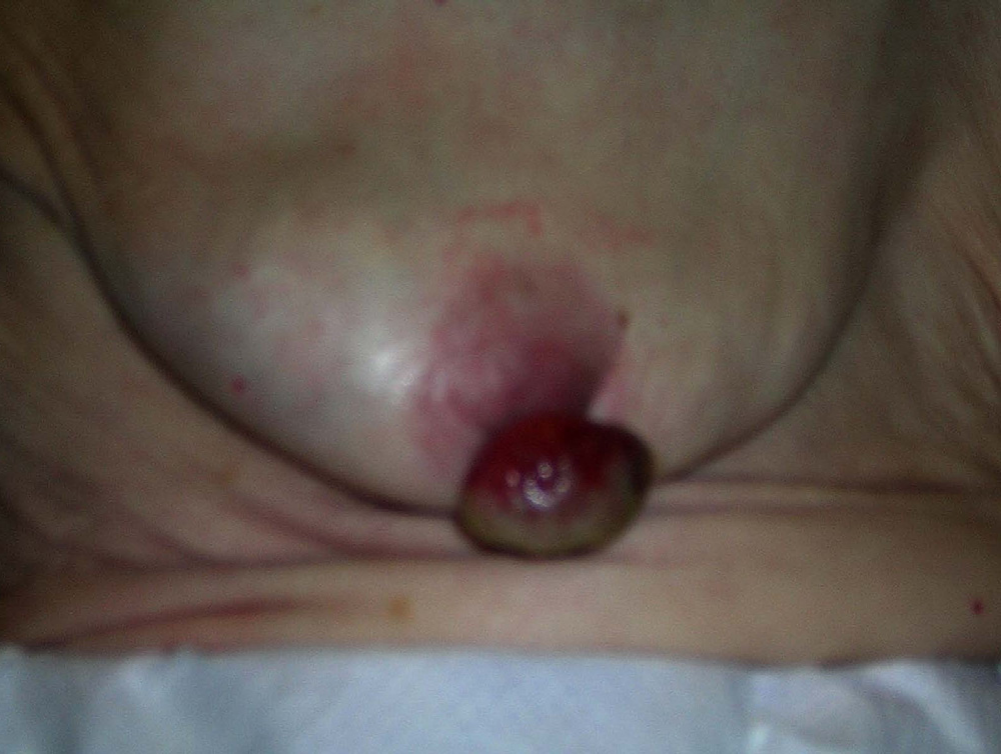
**Apocrine carcinoma of the nipple Image 2**.

She had a polypoid hemispherical growth on her right nipple measuring 40 by 25 mm. This appeared extremely vascular with a necrotic slough covered surface (Figures 1 &2).

She was also found to have a clinically suspicious firm lump measuring 25 by 25 mm in the lower outer quadrant at 8 o'clock position of her right breast 5 cm from the nipple.

There were no palpable masses in her left breast nor palpable lymph nodes in her right axilla. In her left axilla was an enlarged firm lymph node 20 by 20 mm clinically equivocal.

She had bilateral mammography, ultrasound scan of the lower outer quadrant of her right breast and both axilla. Right breast mammography showed a 30 mm radiologically malignant lesion in the lower outer quadrant of her right breast. The left breast showed no suspicious features. Ultrasound scan of the right axilla was normal. Ultrasound scan of her left axilla showed an enlarged lymph node with poor corticomedullary differentiation; radiologically indeterminate.

She had a clinical trucut biopsy of the lump in her right breast and an ultrasound guided trucut biopsy of the indeterminate lymph node in her left axilla. Trucut biopsy of the lump in the right breast lower outer quadrant showed ductal carcinoma in situ. Trucut biopsy of the radiologically indeterminate lymph node in her left axilla showed reactive changes only.

She went on to have right mastectomy with axillary node sampling in the form of sentinel node biopsy.

The lump in in the lower outer quadrant of the breast was found to be predominantly ductal carcinoma in situ of intermediate and high grade of cribriform and apocrine type. Associated with the ductal carcinoma in situ were two small areas of invasive adenocarcinoma. These were grade 3 carcinomas with no lymphovascular invasion, negative oestrogen receptors and Her 2 status negative.

The proximal ducts approaching the nipple were unremarkable. The nipple was entirely replaced by the polypoid lesion. There was too much cytological atypia to consider this as a nipple adenoma. Purely on the basis of cytology and architecture, the appearances were suggestive of ductal carcinoma in situ. There was no evidence of an invasive lesion in this area. The typical features of Paget's disease of the nipple (intra epithelial tumour cells) were not seen. Further immunohistochemistry was performed on the nipple lesion. The tumour cells were negative for oestrogen receptors. Only an occasional smooth muscle cell was demonstrable within the lesion. The appearances therefore argued against a primary lesion of breast epithelium and suggested instead a skin adnexal tumour i.e. apocrine carcinoma.

Two lymph nodes were sampled which were free of tumour.

## Discussion

Apocrine adenocarcinoma is a rare malignant neoplasm arising in areas where there is an abundance of apocrine sweat glands [3]. When it occurs in the breast it is also known as sweat gland carcinoma of the breast. The common site of occurrence is the axilla [4]. They have also been described in other areas which include the scalp, forehead, eyelid, upper lip, sub mandibular skin, chest, pubic skin, nipple, arm, wrist, and finger [3]. Their progression has been found to be variable ranging from weeks to years. Different studies suggest a preponderance in males. Approximately half of patients with apocrine carcinoma have been reported to have regional lymph node metastases at the time of presentation [5]. The highest incidence is between the ages of 40 and 60.

Diagnostic Criteria [6,7] include

At least 75% of microscopic fields must demonstrate the following major criteria

Large cells with abundant eosinophilic cytoplasm

Nucleus to cytoplasm ratio of 1:2 or more

Nuclei round, large and vesicular

Sharply defined cell borders

Minor (non-mandatory) criteria include

Prominent nucleoli in > 50% of fields

Apical cytoplasmic snouts into lumenal spaces

The standard treatment consists of surgery in the form of wide local excision, with or without lymph node dissection.

Apocrine carcinoma responds poorly to chemotherapy. Adjuvant radiotherapy may be used in cases with advanced local or regional lesions.

## Conclusion

There are several pitfalls to be avoided when a patient presents to with a nipple lesion. It is very tempting to consider it as a solely nipple lesion and ignore the breast and axilla. Full breast and axillary examination and triple assessment is necessary to avoid missing any concomitant tumour as in this case.

Majority of nipple lesions are benign and one needs to be aware of malignant lesions of the nipple as these to avoid delay in diagnosis and management.

## Competing interests

The author declares that they have no competing interests.

## Authors' contributions

GA was the only author and has read and approved the final manuscript.

## Consent

Written informed consent was obtained from the patient for publication of this case report and accompanying images. A copy of the written consent is available for review by the Editor-in-Chief of this journal.
